# Cystatin C regulates the cytotoxicity of infection‐induced endothelial‐derived β‐amyloid

**DOI:** 10.1002/2211-5463.12997

**Published:** 2020-10-25

**Authors:** Ron Balczon, Kyle A. Morrow, Silas Leavesley, Christopher M. Francis, Trevor C. Stevens, Ezinne Agwaramgbo, Christopher Williams, Reece P. Stevens, Geri Langham, Sarah Voth, Eugene A. Cioffi, Susan E. Weintraub, Troy Stevens

**Affiliations:** ^1^ Department of Biochemistry and Molecular Biology University of South Alabama Mobile AL USA; ^2^ Center for Lung Biology University of South Alabama Mobile AL USA; ^3^ Department of Cell Biology and Physiology Edward Via College of Osteopathic Medicine Monroe LA USA; ^4^ Department of Chemical and Biomedical Engineering University of South Alabama Mobile AL USA; ^5^ Department of Physiology and Cell Biology University of South Alabama Mobile AL USA; ^6^ Intensive Care and Pulmonology Mayo Clinic Eau Claire WI USA; ^7^ Department of Pharmacology University of South Alabama Mobile AL USA; ^8^ Department of Biochemistry and Structural Biology and Mass Spectrometry Laboratory University of Texas at San Antonio Health Sciences Center TX USA

**Keywords:** Aβ, cystatin C, endothelial cell, pneumonia, *Pseudomonas aeruginosa*, Tau protein

## Abstract

Infection of rat pulmonary microvascular endothelial cells with the bacterium *Pseudomonas aeruginosa* induces the production and release of cytotoxic oligomeric tau and beta amyloid (Aβ). Here, we characterized these cytotoxic amyloids. Cytotoxic behavior and oligomeric tau were partially resistant to digestion with proteinase K, but cytotoxicity was abolished by various denaturants including phenol, diethylpyrocarbonate (DEPC), and 1,1,1,3,3,3‐hexafluoro‐2‐isopropanol (HFIP). Ultracentrifugation for 8 h at 150 000 ***g*** was required to remove cytotoxic activity from the supernatant. Ultracentrifugation, DEPC treatment, and immunodepletion using antibodies against Aβ also demonstrated that cytoprotective protein(s) are released from endothelial cells during *P. aeruginosa* infection. Mass spectrometry of endothelial cell culture media following *P. aeruginosa* infection allowed identification of multiple potential secreted modulators of Aβ, including cystatin C, gelsolin, and ApoJ/clusterin. Immunodepletion, co‐immunoprecipitation, and ultracentrifugation determined that the cytoprotective factor released during infection of endothelial cells by *P. aeruginosa* is cystatin C, which appears to be in a complex with Aβ. Cytoprotective cystatin C may provide a novel therapeutic avenue for protection against the long‐term consequences of infection with *P. aeruginosa*.

## Graphical abstract

Infection of pulmonary endothelial cells by *Pseudomonas aeruginosa* induces the production and release of cytotoxic oligomeric tau and Aβ from the endothelial cells. Cystatin C, which is also secreted by the endothelial cells, can suppress the cytotoxic activity of Aβ. Whether cystatin C directly impacts oligomeric tau remains to be determined. Image produced with BioRender.

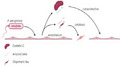

**Abbreviations**
ApoJ: apolipoprotein J
Aβ: beta amyloid protein
DEPC: diethylpyrocarbonate
HBSS: Hank's balanced salt solution
HFIP: 1,1,1,3,3,3‐hexafluoro‐2‐isopropanol
MOI: multiplicity of infection
PA: *Pseudomonas aeruginosa*
PMSF: phenylmethylsulfonylfluoride
PMVEC: pulmonary microvascular endothelial cells
PrP: prion protein
SEM: standard error of the mean

Pneumonia is the second most common infection in hospitals and the most common infection in ICU units [1]. Hospital‐acquired, or nosocomial, pneumonia causes significant morbidity and is the leading cause of death due to nosocomial infections [2, 3]. In addition to these immediate consequences, patients who survive the initial infection and are subsequently discharged from the hospital have elevated death rates in the first year following release from the hospital due to some form of end‐stage organ failure, including cardiovascular, renal, and pulmonary declines, or stroke [4, 5]. Moreover, surviving patients also exhibit decreased cognitive and mental activity [6, 7, 8, 9]. The reasons for these long‐term consequences of nosocomial pneumonia have never been defined.

*Pseudomonas aeruginosa* is one of the major causes of nosocomial pneumonia [10, 11, 12]. *P. aeruginosa* is an opportunistic organism that uses a type III secretion system to transfer various exoenzymes into the cytoplasm of lung cells, where those enzymes ultimately target various membrane phospholipids [13], the actin cytoskeleton [14, 15], and the cytoskeletal protein tau [16, 17, 18]. These events lead to endothelial barrier breakdown in the lung resulting in exudative edema, impaired gas exchange, restrictive physiology, and, oftentimes, death.

Recent studies have provided a potential mechanistic explanation for the long‐term consequences for patients who survive nosocomial pneumonia caused by organisms like *P. aeruginosa*. Specifically, it has been demonstrated that infection of pulmonary endothelial cells in culture, in animal models, and in human patients, leads to the production and release of oligomeric tau and Aβ [18, 19]. These cytotoxic amyloids exhibit characteristics of self‐replicating prion molecules [19], and their release into the blood following infection may provide an explanation for the elevated death rates and memory defects in patients who were successfully treated with antibiotics during the initial hospital stay. According to this scheme, the long‐lived, self‐replicating Aβ and tau would be capable of being transported throughout the body where they could subsequently re‐infect the surviving patient's cells leading to organ failure sometime following hospital discharge. Support for this possibility was shown in studies in which cell culture supernatants generated following infection of cultured endothelial cells, as well as cerebrospinal fluid from pneumonia patients, were able to disrupt long‐term potentiation in rat brain slices [20] and memory in treated animals [21]. Experiments in progress are further investigating the possibility that infection‐induced lung‐derived Aβ and oligomeric tau are transported throughout the circulation leading to poor patient outcomes.

The studies in this paper were designed to allow biochemical characterization of the toxic Aβ and oligomeric tau produced following infection of endothelial cells by *P. aeruginosa*. As will be shown, the cytotoxic amyloids produced following infection are susceptible to various protein denaturing treatments, although pulmonary endothelial cell generated oligomeric tau was relatively resistant to degradation by proteinase K. Moreover, evidence will be presented demonstrating that a cytoprotective regulator of Aβ, cystatin C, is also produced during these infections. Regulation of cystatin C may provide a potential clinical avenue for treating infections caused by *P. aeruginosa*.

## Results

**Fig. 1:**
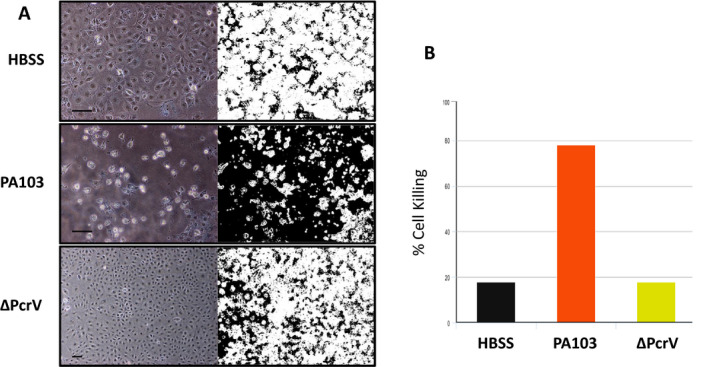
(A) Supernatant collected from *Pseudomonas aeruginosa*‐infected rat PMVECs contains cytotoxins. PMVECs were inoculated with either PA103 or ΔPcrV strain of *P. aeruginosa* and supernatants were collected and processed as described in the Methods section. Supernatant collected from cells infected with PA103 bacteria induced cell killing within 21 h of treatment while supernatant obtained from ΔPcrV treated cells had no effect. Cells treated with HBSS alone were used as a negative control. The images on the left of each paired sample show the phase contrast micrograph and the panels on the right show the imagej‐converted sample used for quantitation of cell killing (see Methods). Bar = 50 μm. (B) The direct quantitation of cell killing for the images shown in Part A using imagej software.

Infection of cultured rat pulmonary microvascular endothelial cells (PMVECs) with *P. aeruginosa* strain PA103 induces the production and release of cytotoxic Aβ and oligomeric tau which exhibit prion characteristics [18, 19]. The basic assay used for analysis of cytotoxic Aβ and tau involves incubating endothelial cells with an infectious strain of *P. aeruginosa* in Hanks Balanced Salts Solution (HBSS), collecting the supernatant 4–5 h after infection, filter sterilizing to remove bacteria, adding the sterile supernatant to confluent cultures of naïve PMVECs and then assessing cell killing 21–24 h later (Fig. 1). Cell killing then is quantified directly by use of imagej software (National Institues of Health, Bethesda, MD, USA) to determine percent area of the culture dish devoid of cells after supernatant treatment (Fig. 1). Supernatant generated following inoculation of PMVECs with ΔPcrV, a strain that lacks a functional type III secretion system, does not contain secreted cytotoxic Aβ and oligomeric tau whereas supernatant produced using strain PA103 does contain the cytotoxic forms (Fig. 1) [19, 20, 21].

This basic assay was then used to assess the stability of Aβ and oligomeric tau cytotoxins following various treatments. For these experiments, supernatant generated following infection of PMVECs with PA103 bacteria was treated with a reagent and then the treated supernatant was added to naïve PMVECs. The amount of cell killing in the treated supernatant was compared to the level of cytotoxicity in untreated PA103 supernatant. Specific supernatant treatments included Proteinase K, HFIP, DEPC, and phenol. Previously it had been shown that cytotoxic amyloids generated during infection of PMVECs by *P. aeruginosa* were resistant to digestion by trypsin for 30 min and were sensitive to treatment with HFIP when HFIP was added at a 10× excess of HFIP to supernatant [19]. These responses were investigated in more detail in initial studies.

**Fig. 2:**
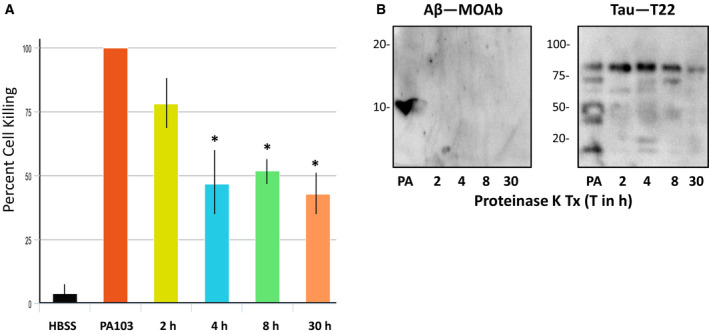
Analysis of Proteinase K‐treated PA103 supernatant. (A) Supernatant obtained from PA103 infected PMVECs was treated with 100 µg·mL^−1^ Proteinase K for 0 (untreated control), 1, 4, 8 or 30 h and then added to PMVECs for 21 h. HBSS was added to one group of cells as a negative control. Cell killing was quantified as described, and presented as standard error of the mean (SEM). Data were analyzed by one‐way ANOVA followed by Tukey's *post hoc* analysis. *N* = 3, *P values compared to PA103 control are 0.005, 0.008, and 0.002 for the 4, 8, and 30 h time points, respectively. (B) Immunoblot analyses of Proteinase K‐treated supernatant using antibodies specific for Aβ (MOAb) and oligomeric tau (T22). Protease treatments were for either 1, 4, 8 or 30 h. Untreated PA103 supernatant (PA) was included as a control. Molecular weights are in kDa.

To assess susceptibility to protease, the broad specificity protease proteinase K was substituted for trypsin and the duration of treatment lasted from 1 to 30 h. As shown in Fig. 2, cytotoxic activity of treated supernatants decreased in a linear fashion until about 4‐h treatment duration, after which time only a very slight decrease in cytotoxicity could be detected in the treated samples. To investigate the nature of the protease‐resistant cytotoxic activity, immunoblot analysis was performed to establish whether oligomeric tau and Aβ were resistant to proteinase K treatment. As shown in Fig. 2, Aβ was nondetectable after 1 h of proteinase K treatment. However, oligomeric tau could still be detected after 30 h protease treatment.

**Fig. 3:**
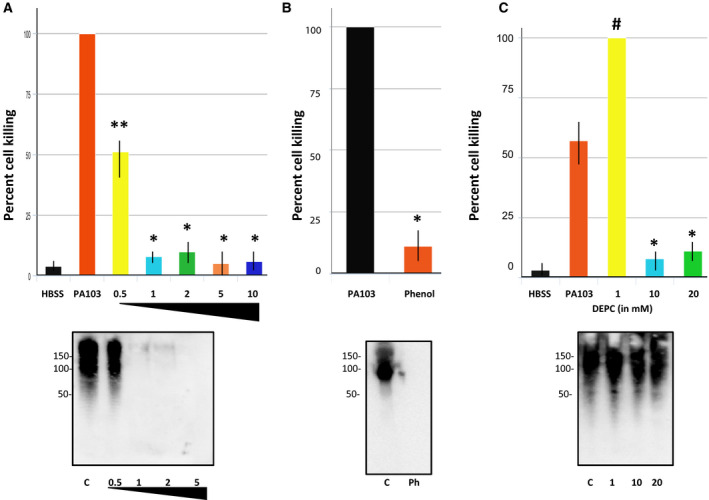
(A) PA103 supernatant was treated with different amounts of HFIP (ratios of PA103 supernatant to HFIP from 1 : 0.5 to 1 : 10) and then cytotoxicity was assayed. Controls included HBSS alone and untreated PA103 supernatant. *N* = 3, ±SEM. **P value compared to PA103 control of 0.008; *P values compared to PA103 control ≤ 0.0001. The corresponding immunoblot of the PA103 supernatant treated with 0.5, 1, 2, and 5 parts HFIP is shown below the cytotoxicity graph. The immunoblot was probed with A11 anti‐amyloid antibody. Untreated PA103 supernatant was included as a control (C). Molecular weights are in kDa. The triangular symbol indicates increasing amounts of HFIP from left to right in both portions of Part A. (B) Untreated (PA103) and phenol‐treated PA103 supernatants were added to cells and cell killing was assessed 21 h later, and presented as ±SEM. Data were analyzed by one‐way ANOVA followed by Tukey's *post hoc* analysis. *N* = 5, *P ≤ 0.00001. Below the graph an immunoblot of untreated PA103 supernatant (C) and phenol‐treated supernatant (Ph) is shown. The immunoblot was probed with A11 anti‐amyloid antibody. Molecular weights are in kDa. (C) PA103 supernatant was treated with either 1, 10, or 20 mm DEPC and then added to PMVECs. Untreated PA103 supernatant and HBSS were used as positive and negative controls, respectively. Cell killing was quantified 21 h after addition of control and treated supernatants, and presented as ±SEM. Data were analyzed by one‐way ANOVA followed by Tukey's *post hoc* analysis. *N* = 3, *P values of relative to control of 0.011 and 0.015 for the 10 and 20 mm samples, respectively; ^#^ *P* value of 0.012 relative to untreated PA103 supernatant. Below the graph, an immunoblot of untreated control PA103 supernatant and supernatant treated with either 1, 10, or 20 mm DEPC is shown. The immunoblot was probed with A11 anti‐amyloid antibody, and molecular weights are given in kDa.

Previous studies assaying HFIP inactivation of cytotoxic Aβ and oligomeric tau generated by PMVECs used a ratio of 10 parts HFIP to 1 part supernatant [19]. Inactivation of endothelial‐derived oligomeric tau and beta amyloid was investigated in a more rigorous manner by performing a dose response using different ratios of HFIP to supernatant. As shown in Fig. 3A, cell killing decreased with treatments of supernatant as low as 0.5 : 1 of HFIP : supernatant, and reached maximal decrease at 1 : 1. No additional decrease in killing was detected at higher ratios of HFIP to supernatant. Previously, we had demonstrated that treatment with a 10‐fold excess of HFIP to PA103 supernatant resulted in loss amyloid epitopes [19]. To assess the effects of HFIP on the structure of endothelial‐derived toxic amyloids at lower ratios of HFIP to supernatant, immunoblot analysis was performed using A11 anti‐amyloid antibody, which we have shown previously recognizes both endothelial‐derived Aβ and oligomeric tau [20, 21]. As shown in Fig. 3B, amyloid characteristics of endothelial‐derived Aβ and oligomeric tau were nearly completely abolished at treatments as low as 1 part HFIP to 1 part supernatant.

Subsequently, the susceptibility of oligomeric tau and Aβ to additional protein denaturants was assessed. Specifically, cell extracts were treated with either phenol or DEPC, and then cytotoxic behavior of the residual supernatant was assessed. As shown in Fig. 3B, phenol treatment abolished cytotoxic activity. Immunoblot analysis using A11 anti‐amyloid antibody demonstrated that phenol treatment nearly completely disrupted the amyloid structure of oligomeric tau and Aβ (Fig. 3B). However, somewhat different results were noted following treatment with DEPC. As shown in Fig. 3C, an increase in cytotoxic activity was noted after treatment of supernatants with 1 mm DEPC. Treatment with higher concentrations of DEPC (10 and 20 mm) completely abolished cytotoxic behavior. Immunoblot analysis using A11 anti‐oligomer antibody demonstrated that amyloid characteristics of Aβ and oligomeric tau were retained after DEPC treatment. As DEPC disrupts protein function by modification of amino acid side chains, these results demonstrate that both amyloid structure and amino acid side chain characteristics are critical for cytotoxic activity of endothelial‐derived Aβ and oligomeric tau.

**Fig. 4:**
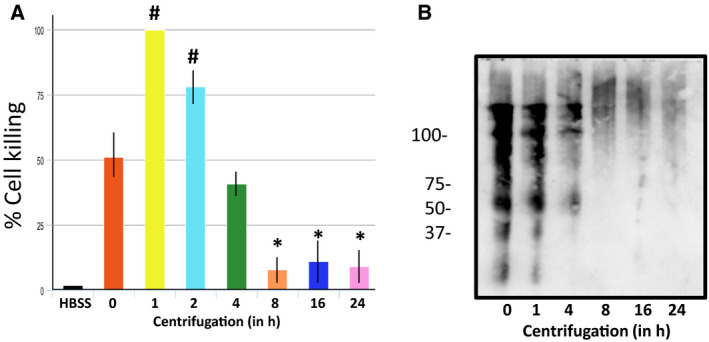
F Cytotoxic amyloids can be removed by ultracentrifugation. (A) Cytotoxic supernatant from PA103 infected PMVECs was centrifuged at 150 000 ***g*** for 1, 2, 4, 8, 16, or 24 h, the supernatants were collected and then applied to PMVECs. Cell killing was quantified 21 h following addition of the centrifuged supernatant to cells. Untreated supernatant (*T* = 0) and HBSS were used as positive and negative controls, respectively. Data were analyzed by one‐way ANOVA followed by Tukey's *post hoc* analysis. *N* = 5, ±SEM. ^#^ *P* values compared to *T* = 0 control of 0.001 and 0.043 for *T* = 1 and 2 h, respectively; *P values compared to *T* = 0 control of 0.003, 0.006, and 0.004 for *T* = 8, 16, and 24 h, respectively. (B) Centrifuged supernatants were analyzed by immunoblot using A11 anti‐amyloid antibody. Residual supernatants after 1, 4, 8, 16, and 24 h of centrifugation are shown. PA103 supernatant that was not centrifuged (*T* = 0) was included as a control. Molecular weights are in kDa.

Studies then were performed to investigate whether cytotoxic Aβ and oligomeric tau were large enough to be pelleted by ultracentrifugation. For these investigations, supernatant obtained from *P. aeruginosa*‐infected PMVECs was centrifuged at 150 000 ***g*** and aliquots were collected at 1, 2, 4, 8, 16, and 24 of centrifugation. The individual supernatants then were applied to cultured PMVECs, and cytotoxicity was assessed. As shown in Fig. 4A, there was an initial increase in cell killing followed by a rapid decrease in cytotoxic activity which reached maximal depletion by 8 h of centrifugation. Immunoblot analyses of the residual fractions following centrifugation was also performed using the A11 anti‐amyloid antibody. As shown in Fig. 4B, a significant portion of the A11 reactive Aβ and oligomeric tau were removed during the first hour of centrifugation at 150 000 ***g***, with additional residual amounts being gradually lost as the length of centrifugation increased.

**Fig. 5:**
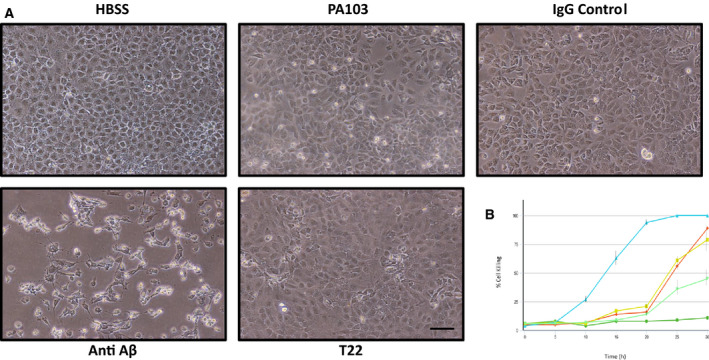
*5*Depletion of cytotoxic supernatant using Aβ antibody increases cell killing. (A) PMVECs were incubated with either HBSS, untreated PA103 supernatant, PA103 supernatant incubated with rabbit IgG followed by Protein A agarose beads (IgG Control), or supernatant that was immunodepleted using anti‐Aβ or T22. The cells then were photographed at different time points and analyzed. As shown, by 10 h treatment, a time where cytotoxicity had not yet been initiated in untreated control PA103 supernatant, considerable cytotoxic activity was noted in the Aβ depleted supernatant. Bar = 50 μm. (B) Quantitation of cell killing in the five groups shown in Part A at different times following addition of supernatant; dark green = HBSS control, red = PA103 supernatant, yellow = IgG control treated supernatant, light blue = Aβ‐depleted supernatant, and light green = T22 (tau)‐depleted supernatant (*N* = 3).

**Fig. 6:**
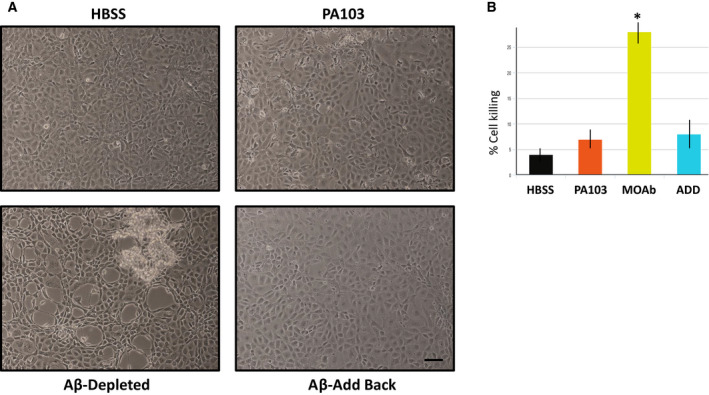
Add back of eluted Aβ to Aβ‐depleted supernatant restores cytoprotective characteristics. (A) PMVECs were treated for 10 h with either HBSS, untreated PA103 supernatant, supernatant that was immunodepleted using Aβ antibody, or Aβ‐treated supernatant which was reconstituted with proteins that were eluted from protein A agarose beads used for immunodepletion. Bar = 50 μm. (B) Quantitation of cell killing at the 10 h time point in the four groups shown in Part A, and presented as ± SEM. Data were analyzed by one‐way ANOVA followed by Tukey's *post hoc* analysis. *N* = 3, *P value of 0.002 compared to untreated PA103 supernatant.

The previous experiments suggested that cytoprotective proteins are released from endothelial cells during infection by *P. aeruginosa* strain PA103, and that these cytoprotective proteins are removed during the first hour of ultracentrifugation at 150 000 ***g*** (Fig. 4) and by treatment with 1 mm DEPC (Fig. 3). To investigate whether Aβ or oligomeric tau were involved as cytoprotective agents, immunodepletion experiments were performed. Specifically, PA103 supernatants were immunodepleted using either anti‐Aβ (MOAB) or anti‐oligomeric tau (T22). The depleted supernatants were then added to PMVECs and cell killing was assessed. As shown in Fig. 5, immunodepletion of Aβ increased cytotoxicity of PA103 supernatant as evidenced by the time of onset of cell killing being decreased to 10 h after addition of the depleted supernatant to cultured cells, a time point at which no cell killing was observed in cultures treated with control PA103 supernatant (Fig. 5). A time‐course analysis determined that the onset of killing and the attainment of maximal killing both occurred about 10 h earlier in the Aβ depleted supernatant than in untreated control PA103 supernatant (Fig. 5). The rapid time of onset of cell killing was specific for depletion of Aβ as it was not observed following depletion of oligomeric tau. Recovery of Aβ from agarose beads and addition of the immune‐isolated Aβ to Aβ immunodepleted supernatant reconstituted a relative cytoprotection of the PA103 supernatant, with no cytotoxicity apparent in cultures at 10 h following addition of the eluted Aβ (Fig. 6).

**Fig. 7:**
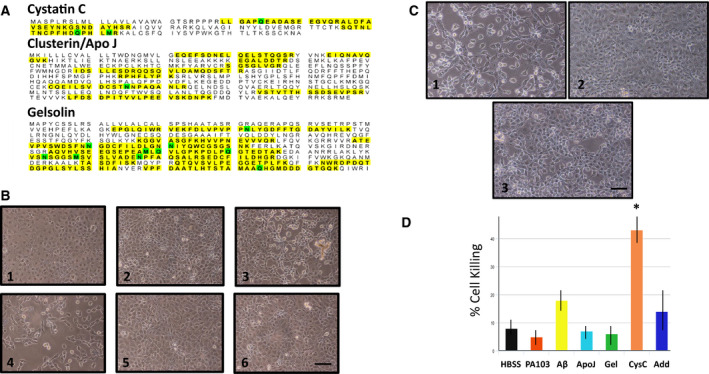
(A) Mass spectrometry of PA103 supernatant identified three potential regulators of beta amyloid toxicity. Identified peptides are highlighted. (B) Immunodepletion of cystatin C increases cytotoxicity of PA103 supernatant. PA103 supernatant was immunodepleted with antibodies against either cystatin C (4), gelsolin (5), or Apo J/clusterin (6). The depleted supernatants were then added to PMVECs and cells killing was assessed at 7 h after addition. HBSS (1), PA103 untreated supernatant (2), and Aβ‐depleted supernatant (3) were used as controls. Heightened cytotoxicity was detected in the cystatin C‐depleted supernatant. Bar = 50 μm. (C) Add back of isolated cystatin C to cystatin C‐depleted supernatant partially rescues cytotoxic characteristics of PA103 supernatant. PMVECs were treated with either supernatant that was immune‐depleted of cystatin C (1), untreated control PA103 supernatant (2), or anti‐cystatin C‐treated supernatant that was reconstituted with proteins that were eluted from agarose beads used for immunoprecipitation (3). Bar = 50 μm. (D) Direct quantitation of cell killing at 7 h after addition of control and immunodepleted supernatants, as well as a cystatin C‐depleted supernatant that was reconstituted with cystatin C that was eluted from beads following immunodepletion (Add). HBSS and untreated PA103 supernatant served as controls. Data were analyzed by one‐way ANOVA followed by Tukey's *post hoc* analysis. *N* = 3, ±SEM. *P value of 0.028 compared to control PA103 supernatant.

Previous published studies have identified an entire cohort of interacting proteins that modulate Aβ toxicity [22]. Mass spectrometry of PA103 supernatant was performed to determine whether any of these reported modulators are released from endothelial cells during infection processes as co‐immunoprecipitation of one or more of these proteins may explain why depletion of Aβ led to increased cytotoxic activity. As shown in Fig. 7A, mass spectrometry analyses identified three known negative regulators of Aβ action, including Apolipoprotein J/clusterin [23, 24, 25, 26, 27], cystatin C [28, 29], and gelsolin [30, 31]. Subsequently, each protein was individually immunodepleted from PA103 supernatants and then the depleted supernatants were added to PMVECs. As shown in Fig. 7B, depletion of neither gelsolin nor Apolipoprotein J/clusterin had any effect on the cytotoxic activity of PA103 supernatant. In contrast, depletion of cystatin C converted PA103 supernatant to a form in which cell killing occurred as early as 7 h after addition of depleted supernatant to PMVECs (Fig. 7B). Direct quantitation of cell killing at the 7 h time point confirmed that depletion of cystatin C increased the cytotoxic activity of supernatant (Fig. 7D). Elution of cystatin C from beads and addition of the eluate to cystatin‐depleted supernatant was able to suppress the enhanced cytotoxic activity of immunodepleted PA103 supernatant (Fig. 7C,D).

**Fig. 8:**
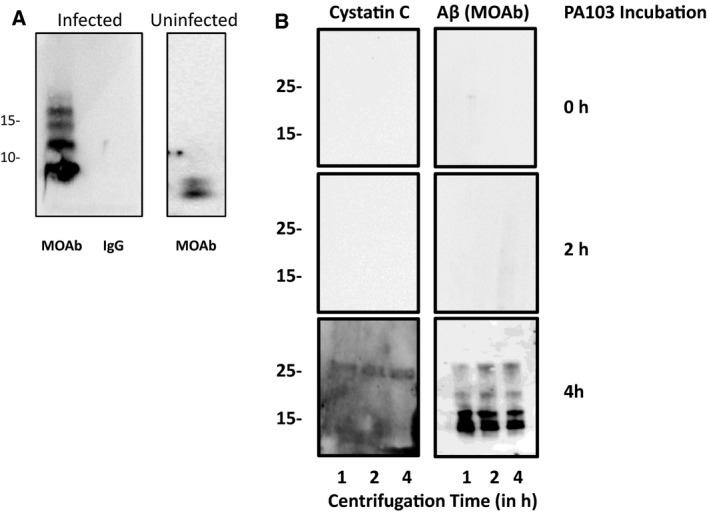
*8*Cystatin C is complexed with Aβ. (A) Co‐immunoprecipitation of cystatin C and Aβ was assessed. Supernatant was collected from control cells that were cultured in HBSS for 4 h (Uninfected) and for cells that were infected with PA103 bacteria for 4 h (Infected). The supernatants were precipitated with either cystatin C antibody or control rabbit IgG. The samples were then analyzed by immunoblotting using MOAB anti‐Aβ antibody. (B) Both Aβ and cystatin C were detected in pellets following ultracentrifugation of PA103 supernatant. Supernatants were collected either immediately after addition of PA103 bacteria (0 h) or after treatment with bacteria for 2 or 4 h. Each supernatant was centrifuged at 150 000 ***g*** for 1, 2, or 4 h and the pelleted material was analyzed using antibody against cystatin C and Aβ (MOAB antibody). Molecular weights are given in kDa.

To establish whether cystatin C and Aβ were complexed, co‐immunoprecipitation analyses were performed. For this, cystatin C was immunodepleted from PA103 supernatant and from culture supernatant derived from uninfected control cells, and then the beads were probed for co‐immunoprecipitation using antibody against Aβ. As shown in Fig. 8A, oligomeric Aβ species in the range of 10–20 kDa were detected in association with cystatin C in supernatants derived from PA103 infected cells, while lower molecular weight forms of Aβ were complexed to cystatin C in supernatants obtained from uninfected cells. Finally, to assess formation of the cystatin C/Aβ complex following infection, PMVECS were treated with PA103 strain of *P. aeruginosa* for either 0, 2, or 4 h, and the individual supernatants were collected. PA103 supernatants were subjected to ultracentrifugation for 1, 2, and 4 h, and the pellets were analyzed for Aβ and cystatin C by immunoblotting. As shown in Fig. 8B, neither cystatin C nor Aβ were pelleted from supernatants derived from cells in which PA103 was added to PMVECs and then immediately collected for analyses (*T* = 0) demonstrating that any cystatin C/Aβ complexes that may have been present in cell culture medium was successfully removed by rinsing prior to addition of bacteria to cells. Moreover, neither cystatin C nor Aβ was present in pellets derived from supernatants collected from cells that were treated with PA103 for 2 h indicating either that Aβ and/or cystatin C had not yet been released from PA103 infected cells or that complexes which formed between the two proteins were not large enough to be pelleted (Fig. 8B). However, when supernatants derived from cells that had been treated with PA103 for 4 h were analyzed, both cystatin C and Aβ pelleted during the first hour of centrifugation at 150 000 ***g*** for 1 h. The size of the pelleted cystatin C was 26 kDa, which is the reported size of cystatin dimers [32, 33, 34].

## Discussion

Various assays were performed to determine the biochemical stability of Aβ and oligomeric tau generated by rat PMVECs following infection with *P. aeruginosa*. Central to the characterization studies was a rapid cell culture cytotoxicity assay in which cell killing could be assessed in 21–24 h. Major findings are that oligomeric tau cytotoxins produced during the infection process are relatively resistant to protease degradation using proteinase K, but both oligomeric tau and Aβ are susceptible to neutralization by HFIP, phenol, and DEPC. In addition, ultracentrifugation established that cytotoxic Aβ and oligomeric tau were large enough to be pelleted. Immunodepletion, DEPC treatment, and centrifugation provided evidence for the presence of a cytoprotective protein in the PA103 supernatant. This cytoprotective protein was identified as cystatin C, and additional studies determined that cystatin C complexes with Aβ. Finally, cytotoxicity was still evident in supernatants depleted of Aβ indicating a critical role for oligomeric tau as an endothelial‐derived cytotoxin.

In a previous study [19], we reported that cytotoxic Aβ and oligomeric tau, which exhibited prion‐like properties, are liberated from rat PMVECs during infection by *P. aeruginosa* bacteria. Previous preliminary characterization of the endothelial‐derived forms of Aβ and tau demonstrated that they were resistant to digestion by trypsin and were not denatured by boiling. In addition, the cytotoxins demonstrated prion‐like behavior as defined by transmissibility during passage between cells in the absence of bacteria. Experiments reported here expanded these previous observations.

Endothelial‐derived cytotoxic Aβ and oligomeric tau were sensitive to various protein denaturants including HFIP, phenol, and DEPC (Figs 2 and 3). However, the mechanisms of inactivation appear to be different. Whereas both phenol and HFIP caused gross rearrangements of oligomeric tau and Aβ as evidenced by loss of immunoreactivity when reacted with A11 anti‐oligomer antibody, DEPC did not reduce reactivity toward the A11 antibody. Rather than cause denaturation of proteins, DEPC modifies amino acid side chains. This result indicates that both amyloid structure and side chains are important for the cytotoxic behavior of Aβ and oligomeric tau. The importance of amino acid side chains and their modifications for assembly of oligomeric tau complexes has recently been demonstrated [35], and these studies further support a role for the importance of amino acid side chains for oligomeric tau toxicity.

Aβ was rapidly digested with proteinase K (Fig. 2) as demonstrated by immunoblot analysis. In contrast, the endothelial oligomeric tau was resistant to digestion by proteinase K with degradation and loss of cytotoxic behavior not being apparent even after incubation for 30 h with 100 µg·mL^−1^ proteinase K. This contrasts to the well‐characterized PrP^sc^ which can be completely proteolyzed by proteinase K between 2 and 4 h of treatment [36]. The reasons for this difference are not clear at present. Regardless, these results demonstrate that oligomeric tau is cytotoxic in the absence of Aβ under the experimental conditions used.

The studies reported here present evidence of an endothelial‐derived cytoprotective molecule being secreted during *P. aeruginosa* infection. This conclusion is based on ultracentrifugation studies, in which cytotoxic activity increased after one hour of centrifugation at 150 000 ***g*** (Fig. 4), by DEPC treatment, in which cell supernatants treated with low concentrations of DEPC exhibited increased cytotoxicity (Fig. 3), as well as immunodepletion studies (Fig. 5). Collectively, these data indicate that cytoprotective protein(s) are released from endothelial cells. The data presented here indicate that cystatin C is the protein responsible for modifying the cytotoxic behavior of endothelial‐derived beta amyloid. At present, it is not possible to state whether cystatin binds to all forms of beta amyloid or only a subset.

Although Aβ is classically considered to be a deleterious molecule due to its link to Alzheimer's disease and other neurologic conditions, recent evidence has indicated that Aβ may have additional functions. For example, it has been shown that Aβ is part of the innate immune response and can act as an anti‐microbial peptide [37, 38]. Our results demonstrate that different forms of Aβ associate with cystatin C, dependent upon whether the Aβ was secreted from uninfected cells or from PMVECs that had been infected by *P. aeruginosa* (Fig. 8), with cystatin C binding to lower molecular weight forms secreted from uninfected cells while being associated with higher molecular weight complexes released from PA103 infected cells. A potential explanation for this varied binding response is provided by our recent studies which demonstrated that infection regulates the type of amyloid secreted from PMVECs [39]. Infection by strains of *P. aeruginosa* that lack a functional type III secretion system or produce catalytically inactive effector exoenzymes leads to the release of noncytotoxic forms of Aβ whereas infection with strains that contain functional type III secretion system and active effector enzymes leads to the conversion of Aβ into cytotoxic forms. The behavior of the type III secretion system and exoenzymes may also help to explain why pelleting of cystatin C and Aβ was only detected when supernatant collected following 4 h infection was analyzed, and not when supernatant collected after 2 h of infection was subjected to ultracentrifugation (Fig. 8). The absence of complexes large enough to be pelleted at early time points may be a consequence of the amount of time required for the infected PMVECs to respond to the infection [40, 41]. Further studies will be required to firmly establish the varied cystatin C/Aβ interactions that occur following *P. aeruginosa* infection.

The cytotoxic forms of Aβ and oligomeric tau present in PA103 supernatant were large enough to be pelleted during ultracentrifugation, and cytotoxic activity was lost after 8 h centrifugation at 150 000 ***g***. This is significantly different than the reported sedimentation rate for the PrP^sc^ particle [42] indicating that the endothelial‐derived cytotoxic prions are smaller and/or less dense than the PrP^sc^ agent. The cytoprotective factor, however, is much larger than the cytotoxic forms and sedimented after only 1 h of centrifugation at 150 000 ***g***. This finding suggests that the cytoprotective form of Aβ is either a large oligomeric form of Aβ, that Aβ is complexed with other cellular components, or both. As shown in Fig. 8, at least portion of the Aβ released following *P. aeruginosa* infection of PMVECs is complexed to cystatin C. Previous investigations of cystatin C in Alzheimer's disease have demonstrated that it protects neuronal cells from Aβ toxicity [29]. Cystatin C appears to be working in a similar fashion in this endothelial system to protect against amyloid cytotoxicity. Additional studies are needed to fully characterize the action of cystatin C in pulmonary infections.

The form of cystatin C that pelleted during ultracentrifugation appeared to be a dimer based on molecular weight (Fig. 8). Cystatin C is a protein that can assemble into oligomers and larger amyloids via the process of domain sharing [32, 33, 34, 43, 44, 45]. The dimerization process is accelerated by cell stress, and dimers are prevalent in pathological material [32, 33, 34, 43, 44, 45, 46]. Most likely, infection of rat PMVECs by *P. aeruginosa* strain 103 is inducing a stress that leads to dimerization of cystatin C. Cystatin C is classically considered as a cysteine protease inhibitor, and dimerization is thought to generate a nonfunctional form of cystatin C as crystallization has determined that the enzyme inhibitory domain of the molecule is buried during the dimerization process [33, 34]. The data here suggest that dimerized forms of cystatin may have an important function as an innate defense mechanism by binding to Aβ thereby inhibiting assembly into polymers. This may have one of three consequences. The data support a model in which the complex of cystatin C and Aβ have a direct cytoprotective role and assist cell survival during the infection process by an undefined mechanism. However, in another related scenario, binding to Aβ may inhibit assembly of beta amyloid into large toxic species promoting survival. A third possibility is that binding of cystatin C to Aβ is an adaptive mechanism of the bacterial cells and assists them with avoiding the innate immune response of the cells. Recent data indicate that beta amyloid oligomers are anti‐bacterial in nature [37, 38, 39]. During evolution, the bacteria may have developed a system where they target cystatin C and induce its dimerization so that it inhibits assembly of beta amyloid into an anti‐bacterial species. Studies investigating whether cystatin C protects bacteria from effects of beta amyloid have not been performed. Clearly, additional studies will be needed to establish the significance of the binding of cystatin C to Aβ during *P. aeruginosa* infections.

In summary, cytotoxic Aβ and oligomeric tau liberated from cultured endothelial cells during infection by the bacterium *P. aeruginosa* were analyzed. The cytotoxic activity of the secreted amyloids is abolished by protein denaturants, although the endothelial‐derived oligomeric tau is resistant to protease digestion and capable of cell killing in the absence of Aβ. Importantly, data are presented identifying cystatin C as a regulator of cytotoxic activity of Aβ during infection by *P. aeruginosa*. As such, cystatin C may be a useful therapeutic target for protection against the consequences of pneumonia caused by *P. aeruginosa*. Studies in progress are investigating this possibility.

## Methods

### Bacterial strains

Two strains of *P. aeruginosa* were used in these studies, and both have been described previously [47]. For most experiments, strain PA103, which expresses both exoenzymes ExoT and ExoU and has a functional type III secretion system for transfer of exoenzymes to infected cells, was used. For some experiments, the mutant ΔPcrV was used. This mutant is incapable of transferring exoenzymes to target cells as the PopB/D channel is not formed [48, 49].

### Cell culture and production of cytotoxic supernatants

Primary cultures of rat pulmonary microvascular endothelial cells (PMVECs) were maintained as described previously [50, 51]. To generate cytotoxic supernatants, PMVECs were grown to confluence and then infected with the appropriate strain of bacteria at a multiplicity of infection (MOI) of 20 : 1 using methods that were described previously [17, 19, 49]. Bacteria were diluted in Hanks' Balanced Salt Solution (HBSS) prior to addition to cells, and treatment with the bacteria was for 4–5 h. Culture supernatants were collected, centrifuged at 2000 ***g*** for 10 min at room temperature to remove debris, and filter sterilized through a 0.22 µm filter to remove bacteria.

### Cytotoxicity assay

Cytotoxic supernatant prepared as described above was added to naïve PMVECs, and the cells were placed in a 37 °C humidified CO_2_ incubator for 21 h. Control cells were treated with HBSS alone. The cells then were photographed and cell killing was quantified by using imagej software as described previously [19].

For some experiments, the cytotoxic supernatant was pretreated before being applied to PMVECs. For some studies, supernatant was treated with 100 µg·mL^−1^ proteinase K at 37 °C for 0, 1, 2, 4, 8 24 or 30 h. The protease was then inhibited by addition of a 1 : 1000 dilution of 1 mg·mL^−1^ phenylmethylsulfonylfluoride (PMSF; diluted in MeOH), and the treated supernatants were added to PMVECs. For these studies, control cells were treated with HBSS that contained PMSF. For other investigations, cytotoxic supernatant was treated with 1,1,1,3,3,3‐hexafluoro‐2‐isopropanol (HFIP) at concentrations ranging from 10 : 1 to 0.5 : 1 HFIP to supernatant for 30 min at 37 °C. The treated samples were then dried via centrifugation under vacuum to remove the HFIP. The dried proteins were then resuspended in a volume of HBSS equal to the starting amount of cytotoxic supernatant, and the resuspended material was added to PMVECs. In a separate set of experiments, cytotoxic supernatant was treated with diethylpyrocarbonate (DEPC) at concentrations of 1, 10, and 20 mm for 30 min at room temperature. The DEPC‐treated supernatants then were added to PMVECs. The last set of studies to test stability of the cytotoxic amyloids involved treating cytotoxic supernatant with phenol. For these experiments, supernatant and saturated phenol (pH 7.0) were mixed 1 : 1 and then vortexed for 60 s. The sample was centrifuged, and the protein‐containing layer was collected, dried as detailed above, resuspended in HBSS and then further desalted back into HBSS by centrifugation using a microcentrifugation device with a 3 kDa cutoff (Millipore product #UFC500324, Burlington, MA, USA). The phenol‐treated supernatant then was added to cultured PMVECs to assess killing activity. In each instance, the presence of residual amyloid in the treated supernatant was analyzed by immunoblot analysis using A11 anti‐amyloid antibody (see below).

### Ultracentrifugation

The relative size of the cytotoxin was determined by subjecting the cytotoxic supernatant to ultracentrifugation and then assaying each centrifuged sample for residual cytotoxic activity. For these studies, equal amounts of cytotoxic supernatant were added to centrifuge tubes and then the samples were centrifuged at 150 000 ***g*** at 4 °C. Samples were collected at 1, 2, 4, 8, 16, and 24 h, and the samples were then either applied to PMVECs to assess cytotoxic activity or concentrated using microconcentration devices with 3 kDa cutoffs before being analyzed by immunoblotting (see below). For some experiments, the pelleted material was collected after ultracentrifugation and analyzed by immunoblot.

### Mass spectrometry

Pulmonary microvascular endothelial cells were rinsed extensively with HBSS and then infected with either PA103 or ΔPcrV bacteria at a MOI of 20 : 1 in HBSS. Cells were incubated for 4 h and then the supernatants were collected, centrifuged, and filter sterilized as outlined above. The supernatant was concentrated 100‐fold using a microcentrifugation filtration apparatus with a 3 kDa cutoff [52]. Proteins were identified using methods outlined previously [53]. Specifically, secreted proteins were separated by SDS/PAGE (0.8‐cm separation; Criterion XT Bis‐Tris 12% gel; Bio‐Rad, Hercules, CA, USA). After staining with Coomassie Blue, the protein‐containing region was excised into six slices and the proteins were reduced and alkylated (iodoacetamide) and digested in situ with trypsin (sequencing grade; Promega, Madison, WI, USA). Each digest was analyzed by capillary HPLC‐electrospray ionization tandem mass spectrometry on a Thermo Scientific Orbitrap Fusion Lumos mass spectrometer (Waltham, MA, USA). On‐line HPLC separation was accomplished with a Thermo Scientific/Dyonex RSLC NANO HPLC system: column, PicoFrit™ (New Objective; 75 μm i.d., Woburn, MA, USA) packed to 15 cm with C18 adsorbent (Vydac; 218MS 5 μm, 300 Å); mobile phase A, 0.5% acetic acid (HAc)/0.005% trifluoroacetic acid (TFA); mobile phase B, 90% acetonitrile/0.5% HAc/0.005% TFA; gradient 3 to 42% B in 30 min; flow rate, 0.4 μL·min^−1^. Precursor ions were acquired in the Orbitrap in centroid mode (scan range, *m/z* 300–1500; resolution, 1–200 000); data‐dependent collision‐induced dissociation spectra of ions in the precursor scan were acquired at the same time in the ion trap (‘top speed’; threshold to trigger MS2, 50 000; quadrupole isolation, 0.7; charge states, 2+ to 5+; dynamic exclusion, 30 s; normalized collision energy, 30%). mascot (v2.6.2; Matrix Science, London, UK) was used to search the spectra against the rat subset of the UniProt database [UniProt_Rat 20170117 (31 383 sequences; 17 231 818 residues)] concatenated with a database of common protein contaminants [contaminants 20120713 (247 sequences, 128 130 residues)]. Cysteine carbamidomethylation was set as a fixed modification and methionine oxidation and deamidation of glutamine and asparagine were considered as variable modifications; trypsin was specified as the proteolytic enzyme, with one missed cleavage allowed. Subset search of the identified proteins by X! Tandem, cross‐correlation with the Mascot results and determination of protein and peptide identity probabilities were accomplished by scaffold (v4.8.7; Proteome Software, Portland, OR, USA). The thresholds for acceptance of peptide and protein assignments in Scaffold were 95% and 99%, respectively.

### Immunoblot analysis

Samples were analyzed by immunoblot analysis using previously reported procedures [19]. Primary antibodies used for immunoblot studies included T22 anti‐tau oligomer antibody (Millipore Product #ABN454), A11 anti‐amyloid antibody (StressMarq Product #SPC‐506D, Victoria, British Columbia, Canada), MOAB anti‐Aβ antibody (Novus Bio Product #NBP2‐13075, Littleton, CO, USA), anti‐apolipoprotein J/clusterin antibody (Boster Biologicals Product #PB9575, Pleasanton, CA, USA), anti‐cystatin C antibody (Novus Product #NBP2‐67898) or anti‐gelsolin antibody (Boster Biologicals Product #PB9209).

### Immunodepletion and immunoisolation

Cytotoxic supernatant was depleted of either oligomeric tau or Aβ using previously reported procedures [19]. Specific antibodies used included T22 (anti‐oligomeric tau) and anti‐β‐amyloid (Invitrogen Product #71‐5800, Waltham, MA, USA). For other studies, gelsolin, cystatin C, or apolipoprotein J were depleted using the previously mentioned antibodies. Depleted supernatants were added to cultured cells to assess cytotoxic activity.

For some studies, the antigen bound to the beads was recovered for add‐back experiments. To achieve this, Protein A agarose beads containing bound antibody‐antigen complexes were washed six times with PBS and then once in PBS containing 0.5 m NaCl. The pelleted beads were suspended in 4 m MgCl_2_ in HBSS and the beads were removed by brief centrifugation in a microfuge. The supernatants containing immune‐isolated antigens and their respective antibodies were collected. As the cytotoxins are heat stable whereas antibodies are heat labile, the antibodies could be inactivated by placing the eluted material at 100 °C for 10 min. The samples then were dialyzed against four changes of HBSS (100‐fold excess for 2–4 h per buffer change) and added to PMVECs along with the appropriate antibody‐depleted supernatant to assess whether cytotoxic activity could be restored.

### Co‐immunoprecipitation

To determine whether cystatin C in cytotoxic supernatants was complexed with β‐amyloid, cystatin C was immunoprecipitated as described above. The beads were then rinsed extensively as detailed previously, and then, the presence or absence of co‐precipitating β‐amyloid was assessed by immunoblotting using anti‐β amyloid antibody. For the control, the immunoprecipitation was performed using non‐immune rabbit IgG (Sigma‐Aldrich product #I‐5006, St. Louis, MO, USA).

## Conflict of interest

The authors declare no conflict of interest.

## Author contributions

RB, TCS, KAM, and SEW designed experiments. RB, KAM, SL, TCS, EA, CW, RPS, GL, SV, EAC, and SEW performed studies. CMF assisted all investigators with data analyses. RB and TCS wrote the manuscript, and all contributing authors assisted with editing.

## Data Availability

Proteomics data will be supplied on request.
